# Comparative Metabolic Phenotyping of Tomato (*Solanum lycopersicum*) for the Identification of Metabolic Signatures in Cultivars Differing in Resistance to *Ralstonia solanacearum*

**DOI:** 10.3390/ijms19092558

**Published:** 2018-08-29

**Authors:** Dylan R. Zeiss, Msizi I. Mhlongo, Fidele Tugizimana, Paul A. Steenkamp, Ian A. Dubery

**Affiliations:** Centre for Plant Metabolomics Research, Department of Biochemistry, University of Johannesburg, P.O. Box 524, Auckland Park, Johannesburg 2006, South Africa; dylanzeiss7@gmail.com (D.R.Z.); msizi.mhlongo17@gmail.com (M.I.M.); fideletu@gmail.com (F.T.); psteenkamp@uj.ac.za (P.A.S.)

**Keywords:** LC-MS, metabolomics, multivariate data analysis, *Solanum lycopersicum*, secondary metabolites

## Abstract

Tomato (*Solanum lycopersicum*) is an important dietary source which contains numerous bioactive phytochemicals. Active breeding programs constantly produce new cultivars possessing superior and desirable traits. However, the underlying molecular signatures that functionally describe these traits are yet to be elucidated. Thus, in this study we used an untargeted metabolomic approach to describe differential metabolic profiles of four cultivars described as having high to intermediate resistance to *Ralstonia solanacearum*. Metabolites were methanol-extracted from leaves, stems and root tissues and analyzed by liquid chromatography coupled with high definition mass spectrometry. Multivariate data analysis revealed cultivar-related differential metabolic phenotypes. A total of 41 metabolites were statistically selected and annotated, consisting of amino acids, organic acids, lipids, derivatives of cinnamic acid and benzoic acids, flavonoids and steroidal glycoalkaloids which were especially prominent in the two highly resistant cultivars. Interestingly, the less resistant cultivars had various fatty acid derivatives in root extracts that contributed to the differentiated metabolic signatures. Moreover, the metabolic phenotype of the STAR9008 (8SC) cultivar with intermediate resistance, was characterized by derivatives of cinnamic acids and flavonoids but at lower levels compared to the resistant cultivars. The 8SC cultivar also exhibited a lack of hydroxybenzoic acid biomarkers, which may be attributed to its lower resistance. These metabolic phenotypes provide insights into the differential metabolic signatures underlying the metabolism of these four cultivars, defining their respective phenotypic traits such as their resistance, tolerance or susceptibility to *Ralstonia solanacearum*.

## 1. Introduction

Tomato (*Solanum lycopersicum*) is one of the world’s most valuable agricultural commodities, accounting for approximately 14% of the world’s annual vegetable production [1,2,3]. Tomatoes are a good source of micronutrients for the human diet, containing rich and diverse bioactive phytochemicals—e.g., polyphenols, carotenoids, alkaloids and tocopherols [4,5]—associated with a variety of health benefits [5,6,7]. Furthermore, the tomato is used as model plant in fruit development, phytochemical accumulation and plant breeding research [4,8].

With the increasing world population and rise of crop pathogens, the agricultural sector is faced with the challenge of maintaining high annual tomato yields. Crop improvement programs have mainly focused on the selection and breeding of tomato cultivars with superior traits such as high crop yield, product uniformity, agronomic and technological attributes as well as natural resistance against various abiotic and biotic stresses [2,9,10,11]. However, the molecular landscapes underlying these traits, which are often multigenic, are still to be comprehensively detailed. Furthermore, the link between the differential metabolism of tomato cultivars and traits determining tolerance to diseases is poorly understood. While susceptible cultivars can easily be eliminated in breeding programs, resistant phenotypes based on multigenic traits are more difficult to evaluate, especially if individual cultivars perform differentially under different environmental conditions and in different locations. These traits can be studied using genomic, transcriptomic and proteomic platforms to identify genes and their products conferring pathogen resistance [12]. These technologies have the drawback that variations in the transcriptome or proteome do not always provide an accurate correlation to the phenotype of the crop under study [13,14].

The metabolome represents the ultimate phenotype of cells, deduced from the changes in gene expression and the modulation of protein function, as well as environmental cues. To functionally describe the metabolism of a biological system, metabolomics has proven to be a powerful and indispensable tool, providing comprehensive molecular signatures of the physiological state of a biological system as well as insightful knowledge of specific biochemical processes [15,16,17,18,19,20]. Metabolic phenotyping has the potential to provide insights into the differential metabolic signatures underlying the phenotypic traits defining resistance, tolerance or susceptibility to microbial infection. Metabolomic analyses, bridging the genotype–phenotype gap, could therefore facilitate the selection of superior traits for the improvement of crop breeding [21,22]. Here, an untargeted metabolomics approach based on an ultra-high-performance liquid chromatography coupled with mass spectrometry (UHPLC-MS) analytical platform was applied to describe the differential metabolic profiles of four tomato cultivars differing in levels of resistance to the soil-borne pathogen *Ralstonia solanacearum*.

## 2. Results

While all four tomato cultivars (Table S1) investigated in this study are characterized as exhibiting an intermediate to high level of resistance to *R. solanacearum*, it should be noted that the cultivars may exhibit variability in performance under certain circumstances in different locations. Accordingly, the STAR9001 (1RC) and STAR9006 (6RC) cultivars have been grouped together in a breeding program as exhibiting high resistance, and the STAR9008 (8SC) and STAR9009 (9SC) cultivars as having intermediate resistance/tolerance to the pathogen (http://www.starkeayres.co.za/commercial-vegetable-seed-variety.php?id=19, accessed on 01 March 2017). 

**Figure 1:**
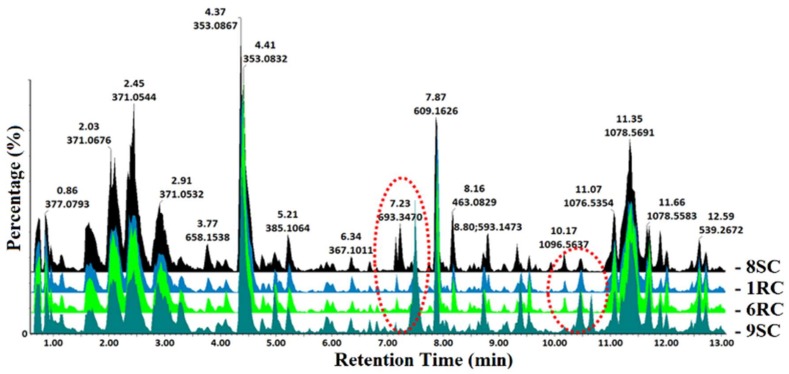
Ultra-high-performance liquid chromatography coupled with mass spectrometry (UHPLC-MS) base peak intensity/ion (BPI) chromatograms (electrospray ionization, ESI(−)) of methanolic leaf extracts from the four tomato cultivars (8SC, 1RC, 6RC and 9SC). The chromatograms show some cultivar-exclusive variations (red dotted circles), reflecting the metabolic differences between the cultivars. Qualitative differences are reflected by the peak intensities, where the *y*-axis represents the relative peak intensity of the metabolite fragments at their respective retention times.

Extracts prepared from roots, stems and leaves of the four tomato cultivars were analyzed on an UHPLC with a quadrupole time-of-flight (qTOF) MS system as detector. Although data was acquired in both positive and negative electrospray ionization (ESI) modes, the analytes ionized better in negative mode, and thus only ESI(−) data is further presented. The representative base peak intensity/ion (BPI) chromatograms (Figure 1, Figures S1 and S2) display the complexity of the tissue extracts obtained from the different cultivars. Visual inspection of the BPI chromatograms shows clear qualitative (presence/absence of peaks) and quantitative variation (differences in peak intensity). 

### 2.1. Differential Metabolic Profiles as Described by Chemometric Models 

Although the chromatographic fingerprints provide a visual description of metabolic differences between samples from different cultivars, informative details are only achieved by data mining and comparative chemometric analyses. Thus, principal component analysis (PCA) allowed exploratory analyses of the data, summarizing the multidimensional data in an intelligible way (by reducing the dimensional space) to detect the underlying characteristics and structures of the data [13,23].

**Figure 2:**
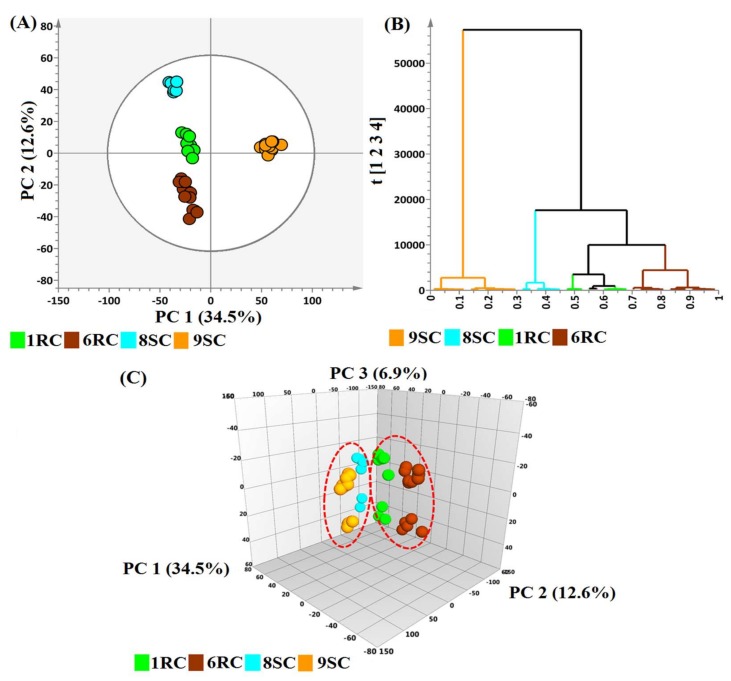
Principal components analysis (PCA) scores plots indicating the general grouping of the variables in the data sets of leaf extracts. 1RC (green), 6RC (red-brown), 8SC (light blue) and 9SC (orange) specify the four cultivars with 1RC exhibiting high resistance and 8SC intermediate resistance/tolerance to *R. solanacearum*. (**A**) The 2D PCA plot of the LC-MS data, from the four cultivars illustrates the general clustering of the variables. The scores plot was computed using the first two principal components (PC1 vs. PC2). The circle in the score plot represents Hoteling’s T2 with 95% confidence interval. (**B**) The hierarchical cluster analysis (HCA) plot shows the hierarchical structure of the data. (**C**) The 3D PCA plot, with the data analyzed in the first three principal components (PC1 vs. PC2 vs. PC3). The red dotted lines indicate distinct clustering of the cultivars.

The computed PCA models, with no overfitting, provided the descriptive assessment of the leaf extract data: the four cultivars were clearly separated in the scores space, as depicted in Figure 2, Figures S3 and S4. The 2D PCA score plots in Figure 2A, Figures S3A and S4A illustrate the distinct clustering of the four cultivars, as well as the general group clustering of the 1RC, 6RC and 8SC cluster and the 9SC cluster. The visualization of the PCA scores plot shows similarities/dissimilarities between (explained by principal component 1 (PC1)) and within (explained by PC2) the sample clusters [20]. The differences in clustering represent the variations seen in the chromatograms in Figure 1. The three-dimensional PCA scores plot in Figure 2C was constructed with the inclusion of the third principal component (PC3) to further explore the patterns in the data, indicated by the dotted red circles, between the resistant (6RC and 1RC) and tolerant (8SC and 9SC) cultivars. The 3D models in Figure 2C, Figures S3C and S4C show the closer grouping of the 8SC cluster to that of the 9SC, rather than with the two resistant clusters (1RC and 6RC); an important observation that could not be seen in the 2D PCA model (Figure 2A). 

Hierarchical cluster analysis (HCA) is a method that builds a hierarchy of the data and projects a dendrogram to represent a hierarchical data structure of the PCA models. The computed HCA plots in Figure 2B, Figures S3B and S4B also indicated that the samples were separated into two major groups (9SC vs. 8SC, 1RC, 6RC) along with a further separation of the second hierarchical clustering (8SC vs. 1RC, 6RC). This suggests that 8SC, although phenotypically classified as tolerant, might share certain metabolic features associated with the resistant cultivars. Orthogonal projection to latent structures discriminant analysis (OPLS-DA) was subsequently applied to the datasets of the 6RC and 9SC cultivars to complement the descriptive information provided by the PCA and HCA models [13,24]. 

**Figure 3:**
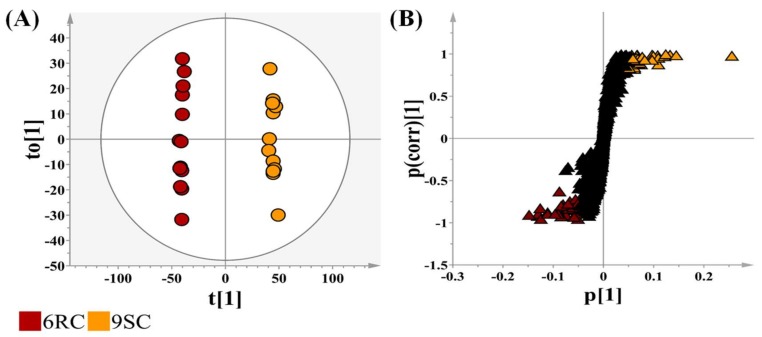
An orthogonal projection to latent structures discriminant analysis (OPLS-DA) model for the data processing of extracts prepared from leaf tissues of two selected cultivars: 6RC (red-brown) and 9SC (orange). (**A**) An OPLS-DA scores plot summarizing the relationship among different datasets to visualize group clustering between the two cultivars. The circle indicates Hoteling’s T2 with 95% confidence interval. (**B**) The corresponding OPLS-DA loadings S-plot. The orange and red-brown triangles indicate statistically significant bio-markers or features identified from the OPLS-DA analysis corresponding to the 9SC and 6RC cultivars respectively. The black triangles indicate features common to both cultivars.

In Figure 3A, the 6RC and 9SC cultivars (furthest apart on the HCA plot, Figure 2B) were compared, with the OPLS-DA scores plot showing distinct sample clustering and clear cultivar separation. The corresponding loadings S-plot (Figure 3B) was used to select discriminating ions between the cultivars. The S-plots allow a visual interpretation of the OPLS-DA models, to facilitate the targeting of statistically significant ions. Discriminating ions with a |p(corr)| of ≥0.5 and a co-variance value of |(p1)| ≥ 0.5 were selected for metabolite annotation using MS spectral-based metabolite identification. The reliability of the models was evaluated with analysis of variance testing of cross validation (CV-ANOVA) as a diagnostic tool, with models of significance having *p*-values of <0.05 [25]. A receiver operating characteristic (ROC) curve in Figure S5D was used to assess the performance of the OPLS-DA models, showing that the computed OPLS-DA models, as binary classifiers, had perfect discrimination with the ROC curve passing through the top left corner to indicate 100% sensitivity and specificity [26]. The predictive capabilities of the OPLS-DA models were validated with the use of a response permutation test (with *n* = 100), shown in Figure S5C [26,27]. The permutation test showed that the calculated models have higher R2 and Q2 values compared to the 100 permutated models, showing that the obtained OPLS-DA models were statistically far better than the generated permutated models.

### 2.2. Metabolic Signatures Differentiating the Four Tomato Cultivars

From the loadings S-plot models, statistically significant ions identified from the methanol extracts of the four tomato cultivars were selected for further analysis, and are presented in Table 1, where the compounds were categorized on the basis of their metabolite class and numbered based on increasing Rt. All compounds present in the tomato tissues, tentatively identified in Table 1, have been previously reported in the literature [4,28,29,30].

All three Venn diagrams in Figure 5 showed minimal metabolite overlap in the tissues (leaf, stems, roots) between the four cultivars, indicating variation at a metabolomic level. The Venn diagram in Figure 5A showed a pattern where many of the glycoalkaloids (#30), flavonoids (#23,26) and cinnamic acids (#7,14,20) (shown in Table 1) are shared in leaf tissues of 1RC, 6RC and 8SC. Table 1 reports a variety of flavonoids, including kaempferol and quercetin derivatives, annotated as markers in the leaf, stem and root tissues of 1RC, 6RC and 8SC, while data analysis of 9SC extracts did not indicate any flavonoid compounds as discriminant ions in the leaves and roots. Figure 5A and Table 1 indicate a caffeoylquinic acid (#10), present only in 1RC leaf tissue as a discriminant ion. An important observation from Figure 5A is that only two biomarkers (#10,16) are shared between the leaf tissues of 6RC and 8SC, with no shared metabolites appearing in the stem and root tissues. Five biomarkers (#20,23,26,29,31) in the leaf tissues and seven metabolites (#11,19,20,21,24,27) in the stems are shared between 1RC and 8SC, which supports the link to the group clustering seen in Figure 2 for the two cultivars. Only three metabolite biomarkers (#6,18,26) were shared in the stem tissues of 1RC and 9SC.

The Venn diagram in Figure 5B shows the general pattern whereby many of the metabolite classes are found in the 1RC stem tissue and shared with the other three cultivars. The 1RC and 8SC cultivars showed the presence of several chlorogenic acid (CGA) derivatives in the stem tissues, shown in Figure 5B, while, conversely, the 6RC and 9SC cultivars lacked CGAs as discriminant ions. The 9SC cultivar contained a number of amino acids (#1,5,6,8) and glycoalkaloid compounds (#30,32–36) as biomarkers in its stem tissue, shown in Figure 5B, but lacked the presence of CGAs and flavonoids as discriminant ions as shown in Table 1. The overlapping quadrant between the 1RC, 6RC and 8SC in the root tissue extracts shown in Figure 5C indicates the presence of caffeoylquinic acid (#14), sinapoylglycoside (#12) and quercetin rutinoside (#25) as shared discriminant ions.

Several lipid metabolites, listed in Table 1, were annotated within the root tissue extracts of 8SC and 9SC, where trihydroxyoctadecanedienoic acid (#38) and hydroxyoctadecanedioic acid (#37,40,41) isomers were identified as discriminant ions. The Venn diagram in Figure 5C indicates that the lipid molecules were mainly associated with the 9SC root tissue, with partial overlap occurring with 8SC root tissue.

The averaged integrated peak areas of selected phenolics in the leaf tissues (presented in Figure 6A,B) revealed concentration differences between flavonoids and hydroxycinnamic acid (HCA) metabolites, with 1RC (resistant) containing the highest integrated peak area composition of CGAs, while 8SC (tolerant) exhibited the highest peak areas of many of the flavonoid compounds. This could indicate that the variability in resistance between 1RC and 8SC may lie with the concentrations of specific intracellular metabolites, rather than the presence or absence of these metabolites. These results also correspond to those shown in Figure 2, further explaining the group clustering of 8SC with the 1RC and 6RC cultivars.

Plots of the average integrated peak areas of the selected metabolites #7, (caffeoylglucaric acid isomer 1) vs. #23 (quercetin hexose deoxyhexose pentoside); #14 (caffeoylquinic acid isomer 2) vs. #26 (quercetin glycoside) from the 1RC, 6RC and 8SC leaf tissues (Figure S6A,B) revealed that the 1RC and 6RC cultivars have similar intracellular concentrations in terms of the combined flavonoid and CGA metabolic pools compared to those of 8SC cultivar. Figure S6C,D indicate clear concentration differences between the metabolites selected from 1RC and 8SC: #22 (feruloylquinic acid isomer 2) vs. #27 (kaempferol rutinoside); #19 (feruloylglycoside) vs. #25 (quercetin rutinoside), to show variability in the combined CGA and flavonoid metabolite pools. Figure S6C shows similarities in integrated peak areas, and thus intracellular metabolite concentrations, of 1RC and 8SC, while conversely, Figure S6D shows that extracts from 1RC had a higher concentration of CGAs, compared to that of 8SC, which in turn, had a higher flavonoid concentration. 

**Figure 4:**
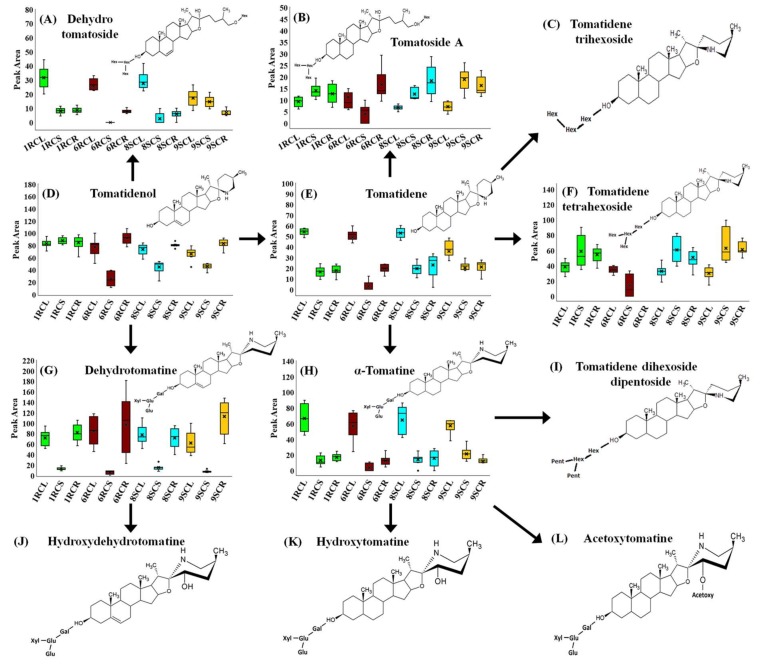
Profiles of the relative quantities of SGAs in methanolic extracts of leaves (L), stems (S) and roots (R) of four tomato cultivars differing in levels of resistance towards *R. solanacearum*. The 1RC and 6RC cultivars are presented by the green and brown bars while the 8SC and 9SC cultivars are indicated by light blue and orange bars respectively. Metabolites not linked to box plots were present in the samples but did not contribute to significant discrimination between the cultivars. (**A**) Dehydrotomatoside, (**B**) tomatoside A, (**C**) tomatidene trihexoside, (**D**) tomatidenol, (**E**) tomatidene, (**F**) tomatidene tetrahexoside, (**G**) dehydrotomatine, (**H**) α-tomatine, (**I**) tomatidene dihexoside dipentoside, (**J**) hydroxydehydromatine, (**K**) hydroxytomatine and (**L**) acetoxytomatine. The model starts with the aglycone tomatidenol, which itself is a cholesterol derivative (not shown in the diagram; adapted from [31]).

**Figure 5:**
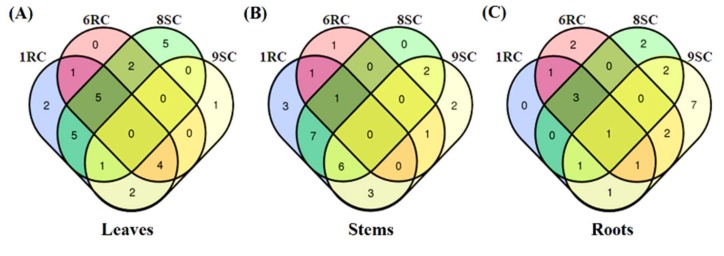
Venn diagrams displaying the partial overlap of statistically significant variables/biomarkers selected from the four OPLS-DA models comparing extracts from (**A**) leaves, (**B**) stems and (**C**) roots of the tomato cultivars (1RC, 6RC, 8SC and 9SC). The numerical values in the diagrams depict the metabolites that are unique to certain cultivars and conversely also shared between the cultivars. (Discriminant ions are listed in Table 1).

**Figure 6:**
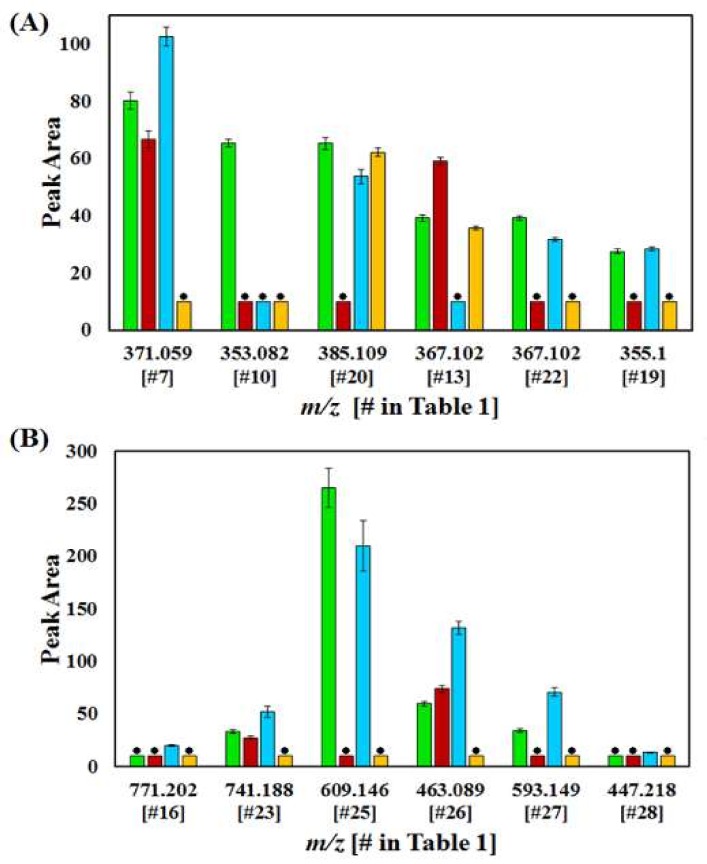
The relative quantification of annotated metabolites representative of the different metabolic pathways in leaf extracts from the four cultivars varying in levels of resistance to *R. solanacearum,* 1RC (green), 6RC (red-brown, 8SC (light blue) and 9SC (orange). (**A**) The cinnamic acid derivatives, identified in each of the cultivars as discriminant ions, e.g., caffeoylquinic acid (#10 *m*/*z* 353.082), feruloylquinic acid (#13 *m*/*z* 367.102), sinapoyl glycoside (#20 *m*/*z* 385.072). (**B**) The flavonoids, identified in each of the cultivars as discriminant ions, e.g., quercetin rutinoside (#25 *m*/*z* 609.146), quercetin glycoside (#26 *m*/*z* 463.089) and kaempferol glycoside (#27 *m*/*z* 593.149). Error bars indicate the standard deviation. Metabolites in (**A**,**B**) not identified as discriminant ions in a selected cultivar are indicated with a black dot above the corresponding bar, with a standard peak area value of 10 (Table 1).

The main steroidal glycoalkaloids (SGAs) detected in the methanolic extracts were dehydrotomatine (#30), present in 1RC, 6RC and 8SC, and α-tomatine (#36), present as discriminant ions only in the two highly resistant cultivars as shown in Table 1. The tomatidenol aglycone (not included in Table 1) showed a higher intracellular concentration in 1RC compared to the other three cultivars (Figure 4D). Tomatidene, as shown in Figure 4E, exhibited a general trend in all the cultivars, with a higher concentration in the leaf tissues compared to stems and root tissues. Dehydrotomatine, as shown in Figure 4G, showed a similar trend across all cultivars where the concentration in the stem tissues was significantly diminished compared to that found in the leaf and root tissues. In contrast, α-tomatine was determined at similar elevated concentrations in the leaf tissues of 1RC and 6RC compared to 8SC and 9SC (Figure 4H). However, this pattern was limited to the leaves as 6RC had a decreased α-tomatine concentration in stem tissues compared to the other cultivars (Figure 4H).

## 3. Discussion

Pathogens often produce a variety of toxic compounds to establish themselves within the host by the disruption of the balanced redox state within the plant metabolome [32]. The host counters the pathogen onslaught with the deployment of chemical defense strategies based on the ability of the plant to synthesize certain defense-related secondary metabolites. In addition to specific phytoanticipins and phytoalexins, plants also utilize metabolites with a more general distribution in its defense arsenal. Plant resistance conferred by HCA derivatives may be linked to a regulated balance between metabolite synthesis and degradation [19]. Lacking a sufficient concentration of HCA derivatives might thus deprive the 9SC cultivar of an added element of resistance against *R. solanacearum*, and subsequently also affects its ability to rapidly produce other phenolic compounds upon infection. These include phenylpropanoids that can undergo multiple transformations for the synthesis of lignin and suberin for reinforcement of cell walls [33,34]. The deposition of lignin in the cell wall strengthens a physical barrier that limits pathogen development while simultaneously also preventing distribution of plant resources to the pathogen, depriving it of essential nutrients [33,35,36].

Defense-related HCA-conjugates include CGAs, which have a contributing role in plant resistance [28,37]. CGAs belong to the family of phytochemical esters produced from the shikimate- and early phenylpropanoid pathways that have been shown to confer plant resistance proportional to their intracellular concentration [20,36,38,39]. CGAs are natural antimicrobial compounds, which allows them to inhibit pathogenic factors and enzymes, thus suppressing pathogen virulence [40]. CGAs confer resistance through the donation of protons for radical reduction to inhibit oxidative reactions involving pathogen toxins [32]. In addition, CGAs, through reverse esterification reactions, can provide an untapped metabolic pool of quinic acid along with derivatised cinnamic acids (HCAs) that can be incorporated into the phenylpropanoid pathway, influencing the metabolic flux when the rapid production of phytoalexins or lignin precursors is required [20,28]. 

Flavonoids constitute the highest percentage of secondary metabolites in tomatoes, with the main sub-classes including flavonols (e.g., kaempferol and quercetin), flavanols (e.g., catechins), flavanones (e.g., naringenin), stilbenes (e.g., resveratrol) and anthocyanidins. In addition to the metabolites’ natural antimicrobial activity, flavonoid structures contain multiple hydroxyl groups which bestow a reducing potential that allows for the rapid neutralization of pathogen-derived free radicals [40,41]. 

As shown in Figure S6C,D, extracts from the tolerant 8SC cultivar contained high levels of both HCAs and flavonoids, similar to that of the resistant 1RC cultivar. In addition, 8SC also contains SGA levels comparable to the other three cultivars throughout all three plant organs, as shown in Figure 4. However, the hydroxybenzoic acid (HBA) derivatives, dihydroxybenzoic acid and benzyl alcohol-hexose-pentoside, were annotated as biomarkers in the various tissues of 1RC, 6RC and 9SC, but not in any tissue-type of 8SC (Table 1). HBA-derivatives have been shown to provide a degree of antibacterial- and antioxidant activity and may also function as precursors in the production of salicylic acid (SA), a known inducer of the systemic acquired resistance (SAR) response in plants [42,43]. Recent data suggest that the glycosylation of dihydroxybenzoic acids plays a previously unrecognized role in the plant innate immune response through modulating SA homeostasis [44]. The low levels of HBA-derivatives synthesized in 8SC tissues may thus be linked with its decreased resistance towards *R. solanacearum*. These findings suggest that the 8SC cultivar displays phenotypic traits of both resistant and intermediate cultivar lines, with an abundance of HCAs and flavonoid biomarkers typically associated with resistant cultivars and a decreased presence of HBA-derivatives. 

Lipids are structural and functional components of membranes as well as alternative energy sources in primary metabolism for organ growth and development [45,46]. As described, the lipid molecules were identified as biomarkers primarily in root tissues of the tolerant 8SC and 9SC cultivars. Lipids are also precursor molecules for the synthesis of various phytohormones, such as the fatty acid-derived jasmonic acid (JA), which itself has known defense gene regulating capabilities [19,45,47,48]. Some linoleic acid precursors, including bioactive oxylipins, have been reported to function as signaling metabolites in addition to their antimicrobial properties, and therefore function as protective compounds in plant organs [40,46,49]. The production of lipid-derived defense-related compounds occurs at a slower rate, compared to that of the cinnamic acids, benzoic acids and flavonoids, and can be suggested to fulfil an alternative secondary defense role upon infection. 

Recent studies have shown that lipids are not only associated with the initiation of plant reactions as defense signaling mediators, but also mitigate various metabolic processes to reduce the severity of environmental stress [46,47]. An example of this would be polyunsaturated fatty acids that attenuate cell damage upon stress caused by reactive oxygen species [46]. Lipid peroxyl radicals can also be produced by the plant through non-enzymatic processes that react with biomolecules in close proximity, altering their natural biological structures, and eventually leading to cell death [48,50].

SGAs are nitrogen-containing steroids, often found in a glycosylated form [51,52,53]. These secondary metabolites serve as phytoanticipins, providing the plant with chemical defenses against a wide range of pathogenic microorganisms [40,54]. SGAs function in plant defense through complex formation with sterols and the disruption of pathogen membranes, which is then followed by the leakage of the contents of pathogen cells and eventual cell death [31,55]. α-Tomatine can diffuse through pathogen membranes with ease but has been shown to have a lower antimicrobial activity compared to that of tomatidene and tomatidenol [55]. It has been suggested that the sugar moiety present in the structure of α-tomatine contributes to the metabolite’s relative stability during the formation of glycoalkaloid-sterol complexes and further increases metabolite solubility for increased distribution through the cell system of the pathogen [55,56,57]. Plant species with high resistance to microorganisms, especially fungal pathogens, often have higher concentrations of SGAs compared to their susceptible counterparts [54], as also observed in this study (Figure 4D,G,H). The elevated concentration of α-tomatine in the cultivars can be speculated to provide a contributing factor to the resistant phenotype.

Figure 4 shows the natural distribution of SGAs throughout the plant, with the highest concentration localized within the leaf tissue. The tomatidene derivatives hydroxytomatine (#29), dehydrotomatoside (#32), tomatidene tetrahexoside (#33) and tomatoside A (#35), shown in Table 1 and Figure 4A,B,I,K, may have similar functions to that of α-tomatine, with the variation in the structure of the sugar moiety better facilitating membrane penetration [56,57]. Furthermore, the steroid moieties, tomatidene and tomatidenol, of these compounds have known allelopathic activity which can alter gene expression to disrupt normal pathogen homeostasis during infection [52,53,56].

## 4. Materials and Methods

### 4.1. Plant Cultivation

Seeds from four tomato cultivars, STAR9001 (1RC), STAR9006 (6RC), STAR9008 (8SC) and STAR9009 (9SC) were used for this study. The tomato seeds were obtained from a breeding program for resistance against *R. solanacearum*; general information relating to cultivar resistance properties can be obtained (Starke Ayres, Pty. Ltd., Bredell, South Africa, http://www.starkeayres.co.za/commercial-vegetable-seed-variety.php?id=19, Supplementary Information). All cultivars were grown in germination mixture (Culterra, Muldersdrift, South Africa). Each cultivar was grown in triplicate under greenhouse conditions: a light/dark cycle of 12 h/12 h, with the light intensity set at 80 µmol/m^2^/s and the temperature regulated to between 22–24 °C. Once the plants reached 8-week maturity, the leaves, stems and roots of each cultivar were harvested, frozen and stored at −80 °C until metabolite extraction.

### 4.2. Metabolite Extraction and Sample Preparation

Tissues frozen with liquid nitrogen were pulverized with a mortar and pestle. Two grams of stem material and 1 g of root material from each cultivar were extracted with 80% methanol in a 1:1 (*w*/*v*) ratio. The samples were sonicated twice in a sonicator bath (Sonopuls, Berlin, Germany) for 30 min at 20 °C. Cell debris was pelleted with a bench-top swinging-bucket centrifuge at 5525× *g* and 5 °C for 20 min. The supernatants were evaporated to 1 mL using a rotary evaporator at 55 °C, carefully transferred into 2 mL micro-centrifuge tubes and dried in a heating block overnight. The samples were then reconstituted in 500 µL of 50% HPLC-grade methanol: MilliQ water solvent (1:1, *v*/*v*). The samples were filtered through 0.22 µm nylon syringe filters into vials fitted with 500 µL inserts and stored at 4 °C until analyzed. Each sample was prepared in triplicate (biological repeats) and subsequently analyzed in triplicate (technical repeats) to gain accuracy and precision (*n* = 9). A pooled sample consisting of aliquots from all the samples was prepared and used as quality control (QC) to monitor the stability of the samples, the instrumentation and analyses. 

### 4.3. Ultra-High-Performance Liquid Chromatography (UHPLC) Analyses

Two microliters of each sample extract was analyzed on an UHPLC system (Acquity, class Classic, Waters Corporation, Manchester, UK). The analytes were separated on an Acquity HSS T3 reverse-phase column (2.1 × 150 mm × 1.7 µm; Waters Corporation, Milford, MA, USA) using a binary solvent system consisting of MilliQ water and acetonitrile (Romil Chemistry, Cambridge, UK), with both solvents containing 0.1% formic acid (Sigma, Munich, Germany). A gradient elution method was used over a 30 min run with a flow rate set to 0.4 mL/min. The elution was started at 2% (*v*/*v*) acetonitrile from 0–1 min, raised to 60% acetonitrile from 1–22 min, taken up to 95% from 22–23 min then kept constant at 95% acetonitrile from 23–26 min. The composition of the mobile phase was then reverted to 2% acetonitrile from 26–27 min, for column cleaning and equilibration from 27–30 min. Chromatographic elution was monitored with a photodiode array (PDA) detection system with a scanning range between 200–500 nm, 1.2 nm bandwidth resolution and a sampling rate of 20 points/s.

### 4.4. Quadrupole Time-of-Flight Mass Spectrometry (q-TOF-MS) Analyses

The metabolites were detected with the aid of a SYNAPT G1 high definition mass spectrometer (Waters Corporation, Manchester, UK) set to acquire data in both ionization operation modes. The MS conditions were as follows: a capillary voltage of 2.5 kV, sample cone voltage of 30 V, microchannel plate detector voltage of 1600 V, desolvation temperature of 450 °C, source temperature of 120 °C, cone gas flow of 50 L/h, desolvation gas flow of 550 L/h, *m*/*z* range of 50–1500, scan time of 0.2 s, interscan delay of 0.02 s, mode set as centroid, lockmass flow rate of 0.1 mL/min, lockmass set as leucine enkephalin (554.2615 Da) and a mass accuracy window of 0.5 Da. High-purity nitrogen was used as the desolvation-, cone- and collision gas. The MS analyses were set to perform unfragmented as well as four fragmenting experiments (MS^E^) simultaneously by collision energy ramping from 10 to 50 eV. Data acquisition at these various collision energies was performed to facilitate metabolite fragmentation for later assistance in downstream structure elucidation and compound annotation [19,29].

### 4.5. Data Analyses

The UHPLC-ESI-MS data sets were analyzed with Markerlynx XS software (Waters Corporation, Manchester, UK). The raw UHPLC-ESI-MS data was processed for matrix generation with MarkerLynx XS^TM^ 4.1 software, with the following parameters: retention time (Rt) range of 0.60–21 min and *m*/*z* mass range of 50–1500 Da. The Rts were allowed to differ by ±0.20 min and the *m*/*z* values by ±0.05 Da. The mass tolerance was 0.01 Da and the intensity threshold was 10 counts. Only the data matrices with noise level less than 50% (MarkerLynx cut-off) were retained for downstream data analyses. The MarkerLynx application uses the patented ApexPeakTrack algorithm to perform accurate peak detection and alignment. Furthermore, MarkerLynx performs sample normalization, based on total ion intensities of each defined peak. Prior to calculating intensities, the software performs a modified Savitzky-Golay smoothing and integration. The obtained data matrices were exported to soft independent modeling of class analogy (SIMCA) software, version 14 (Umetrics, Umeå, Sweden) for multivariate data analysis. Prior to PCA and OPLS-DA modelling, the data were mean-centered and *Pareto*-scaled to put all variables on equal footing, minimize variable redundancy and adjust for measurement errors. A nonlinear iterative partial least squares algorithm (in-built within SIMCA software) was used to handle missing values, with a correction factor of 3.0 and a default threshold of 50%. Two unsupervised methods, PCA and HCA, and a supervised method, OPLS-DA, were employed. The OPLS-DA models were used to compare the 6RC and. 9SC cultivars for the identification of ions responsible for the discrimination between the two groups [13]. A seven-fold cross-validation (CV) method was applied as a tuning procedure in computing the models. Thorough model validation steps were consistently applied; and only statistically valid models were examined and used in data mining for metabolite annotation. Only statistically significant metabolites exhibiting a variable importance in projection (VIP) score >1 (calculated by the SIMCA software) were further investigated.

### 4.6. Metabolite Annotation and Qualitative Comparison

The chemical and structural identities of the metabolites were elucidated using their respective mass spectral patterns obtained during the MS analyses. MS spectral-based metabolite identification was performed based on: sufficient and accurate mass fragment information, accurate calculation of each compound’s elemental composition and database searches for possible metabolite annotation. The putative empirical formula of each statistically significant extracted ion peak (XIC) in the mass spectra was obtained and searched in databases such as ChemSpider (www.chemspider.com, accessed on 5 July 2017) and Dictionary of Natural Products (dnp.chemnetbase.com/, accessed on 5 July 2017) for the identification of possible compound matches [19]. Metabolites were tentatively identified/annotated to level 2 of the Metabolomics Standards Initiative (MSI) [58]. Venn diagrams were then constructed to aid in the further interpretation of the results.

### 4.7. Semi-Quantitative Comparison

Upon identification of statistically significant biomarkers, the relative concentrations of the discriminant ions were calculated in the four cultivars, as represented by peak intensities obtained from the original chromatograms. Differences were indicated by the mean of nine peak intensity values while precision was indicated by the calculated standard deviation. These values were used to construct bar graphs and box-and-whiskers plots as graphical representations showing the relative concentration differences of the statistically selected biomarkers in the tissues of the four cultivars. 

## 5. Conclusions

A variety of tomato cultivars are commercially available with little to no information on the molecular mechanism(s) that enable them to perform better under specific conditions. The multivariate metabotyping results presented here contribute to the understanding of natural variation present in the tomato metabolomes investigated. The identified metabolites were amino acids, organic acids, cinnamic acid derivatives, hydroxybenzoic acid derivatives, flavonoids, steroidal glycoalkaloids and lipids. The differences in the metabolic profiles and metabolites identified in this work indicate that untargeted metabolomics can be used to distinguish between cultivars exhibiting differential levels of resistance to *R. solanacearum*. The results also indicate that the presence or absence of specific metabolites cannot be the only concluding answer, but that relative concentrations or ratios of metabolites also play an important role in constituting the disease-resistant phenotype. The secondary metabolite classes (e.g., HCAs, flavonoids, HBA-derivatives, glycoalkaloids) mentioned in this article play crucial roles in plant defense, with the relative abundance of the metabolites, as phytoanticipins, being linked to each cultivar’s capacity to preferentially utilize the pre-formed resources at its disposal to resist and fend off invading pathogens. Cultivar-specific traits can thus be linked to the regulation of a delicate balance of secondary metabolite combinations of diverse biosynthetic origins as opposed to the regulation of a small number of vital biomarkers. The metabolic components contributing to the underlying physiological and phenotypic traits can be detected and identified using an untargeted LC-MS-based metabolomics approach, which will thus be a huge benefit in improving the understanding of the complexities of plant metabolism. 

## Figures and Tables

**Table 1:** Annotation of the statistically significant secondary metabolites identified within methanolic extracts of leaves (L), stems (S) and roots (R) of the four tomato cultivars exhibiting a spectrum of intermediate to high resistance to *R. solanacearum* (1R, 6R, 8S and 9S). The metabolite ions annotated (Metabolomics Standards Initiative (MSI), level 2) showed strong correlation to cultivar variation. Metabolites present in the tissue of a cultivar as discriminant ions identified through OPLS-DA, are indicated with (o).

#	Rt (min)	Compound Name	Mass *(m*/*z)*	1RC			6RC			8SC			9SC		
				L	S	R	L	S	R	L	S	R	L	S	R
**Amino Acids**			**[M − H]^−^**												
1	0.85	Glutamic acid	146.043											o	
5	1.17	Pyroglutamic acid	128.032					o		o				o	o
6	1.38	Leucine/Isoleucine	130.084		o	o						o		o	o
8	1.90	Phenylalanine	164.070		o						o			o	
24	7.51	Acetyl tryptophan	245.088	o									o		
**Organic Acids**			**[M − H]^−^**												
3	0.97	Malic acid	133.011	o	o			o	o				o		o
4	0.98	Fumaric acid	115.000	o				o	o						o
15	4.77	Citrate pentoside	323.131				o			o					
18	5.07	Ascorbic acid	175.036		o				o		o				
**Cinnamic Acid Derivatives**			**[M − H]^−^**												
2	1.41	Caffeoylglycoside	341.105		o			o		o	o	o			
7	1.64	Caffeoylglucaric acid isomer 1	371.059	o			o			o					
9	2.07	Caffeoylglucaric acid isomer 2	371.054				o			o					
10	2.83	Caffeoylquinic acid isomer 1	353.082	o	o						o				
12	3.98	Sinapoylglycoside isomer 1	385.072							o					
13	4.10	Feruloylquinic acid isomer 1	367.102	o	o		o						o		
14	4.41	Caffeoylquinic acid isomer 2	353.083	o	o	o	o		o	o	o	o			
19	5.07	Feruloylglycoside	355.100	o	o	o			o	o	o	o			o
20	5.21	Sinapoylglycoside isomer 2	385.109	o	o	o			o	o	o	o	o		
21	5.92	Feruloylglucaric acid	385.184										o		o
22	6.36	Feruloylquinic acid isomer 2	367.102	o						o	o			o	
**Hydroxybenzoic Acid Derivatives**			**[M − H]^−^**												
11	3.31	2,5-Dihydroxybenzoic acid pentose	285.055	o	o		o						o		o
17	4.99	Benzyl alcohol hexose pentoside	401.142	o	o	o	o		o				o	o	
**Flavonoids**			**[M − H]^−^**												
16	4.96	Quercetin dihexose deoxyhexoside	771.202							o					
23	7.16	Quercetin hexose deoxyhexose pentoside	741.188	o	o		o			o	o				
25	7.90	Quercetin rutinoside	609.146	o	o	o			o	o		o		o	
26	8.17	Quercetin glycoside	463.089	o	o		o			o	o				
27	8.83	Kaempferol rutinoside	593.149	o	o					o	o			o	
28	9.12	Kaempferol glycoside	447.218							o					
**Steroidal Glycoalkaloids**			**[M + H]^+^**												
29	10.18	Hydroxytomatine	1050.548	o						o					
30	11.07	Dehydrotomatine isomer 1	1032.540	o	o	o	o		o	o	o			o	o
31	11.31	Lycoperoside G/Lycoperoside F/Esculeoside A	1136.560		o	o									o
32	11.43	Dehydrotomatoside	1079.560	o	o		o				o		o	o	o
33	11.43	Tomatidene tetrahexoside	1062.560		o						o			o	
34	11.44	Dehydrotomatine isomer 2	1032.550		o						o			o	
35	11.45	Tomatoside A	1081.570											o	
36	11.66	α-Tomatine	1034.543	o			o				o			o	o
**Lipids**			**[M − H]^−^**												
37	13.92	Hydroxyoctadecanedioic acid	329.230									o			
38	14.00	Trihydroxyoctadecadienoic acid	327.220												o
39	14.35	13-Amino-13-oxo-tridecanoic acid	242.170												o
40	15.00	Hydroxyoctadecanedioic acid	329.230												o
41	16.20	Hydroxyoctadecanedioic acid	329.230									o			o

The steroidal glycoalkaloids highlighted in grey (#29,30,32,33,35 and 36) correspond to the metabolite structures present in the metabolic pathway shown in Figure 4.

## Supplementary Materials

Supplementary materials can be found at http://www.mdpi.com/1422-0067/19/9/2558/s1.
