# Supplementary material for: Comparative Metabolic Phenotyping of Tomato (Solanum lycopersicum) for the Identification of Metabolic Signatures in Cultivars Differing in Resistance to Ralstonia solanacearum
Source: Int J Mol Sci. 2018 Aug 29;19(9):2558. doi: 10.3390/ijms19092558 (PMC6163672; doi:10.3390/ijms19092558)
Supplement: Supplementary file 1 [file ijms-19-02558-s001.pdf]

International Journal of Molecular Sciences

MDPI

Supplementary data

Table S1. *Solanum lycopersicum* cultivar information guide.

Disease Resistance Phenotypes				
	**STAR 9001 (1RC)**	**STAR 9006 (6RC)**	**STAR 9008 (8SC)**	**STAR 9009 (9SC)**
High Resistance	Va:1 / Vd:1 Fol:1-2 / Rs	Va:1 / Vd:1 Fol:1-2 / Rs	Va:1 / Vd:1 Fol:1-2	Va:1 / Vd:1 Fol:1-2
Intermediate resistance	Ma, Mi, Mj,	Ma, Mi, Mj, Lt	Ma, Mi, Mj, Rs, Lt, TSWV	Ma, Mi, Mj, Rs, Lt, TSWV

Abbreviation key:

Scientific Name	Common Name	Abbreviation
Viral Pathogens		
Tomato spotted wilt virus	Tomato spotted wilt	TSWV
Bacterial Pathogens		
Ralstonia solanacearum	Bacterial wilt	Rs
Fungal Pathogens		
Fusarium oxysporum f.sp. *lycopersici*	Fusarium wilt	Fol
Leveillula taurica	Powdery mildew	Lt
Verticillium albo-atrum	Verticillium wilt	Va
Verticillium dahliae	Verticillium wilt	Vd
Nematode Pathogens		
Meloidogyne arenaria	Root-knot	Ma
Meloidogyne incognita	Root-knot	Mi
Meloidogyne javanica	Root-knot	Mj

*The above information is provided and can be obtained on the Stark Ayres online webpage: https://www.starkeayres.co.za/com_variety_docs/Tomatoes-Determinate-varieties-Crop-Table-Website.pdf

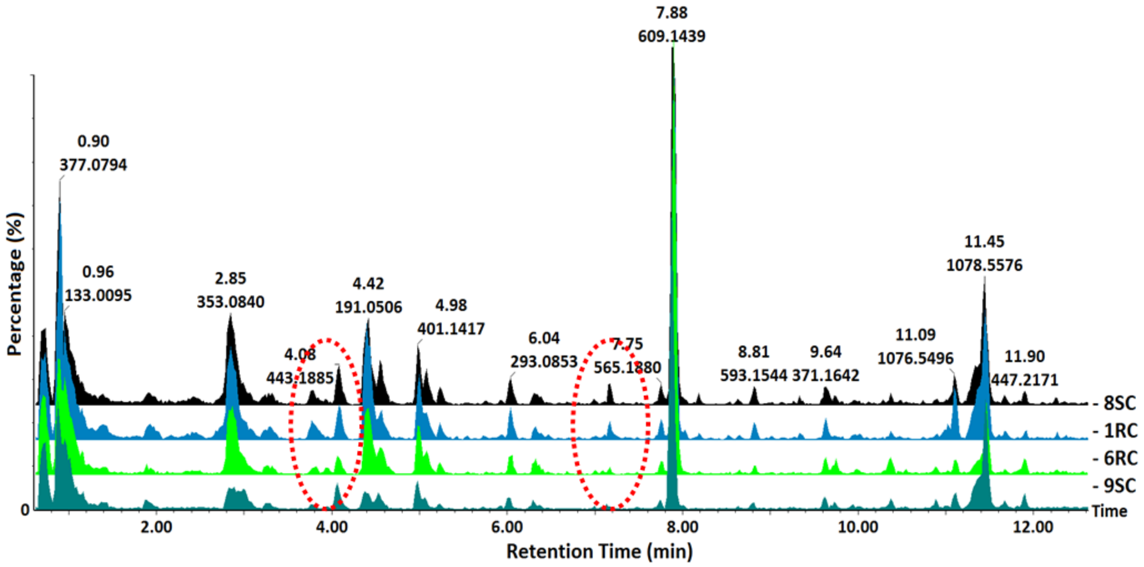

Figure S1. UHPLC-MS BPI chromatogram overlays of methanolic extracts from <u>stem</u> samples of the four tomato cultivars (8SC, 1RC, 6RC and 9SC – Table S1). The chromatograms were constructed and overlayed using Markerlynx™ software as a tool for visual comparison. The *y*-axis represents the relative peak intensity of the metabolite fragments at their respective retention times. The chromatograms show some time-dependent variations (indicated with the red circles), providing an indication to the variations in the individual metabolomes.

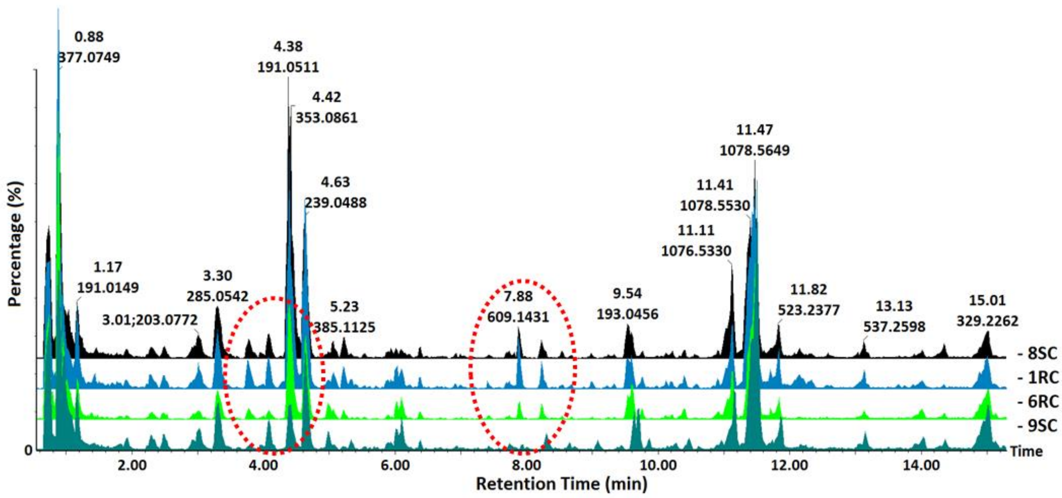

Figure S2. UHPLC-MS BPI chromatogram overlays of methanolic extracts from <u>root</u> samples of the four tomato cultivars (8SC, 1RC, 6RC and 9SC – Table S1). The chromatograms were constructed and overlayed using Markerlynx™ software as a tool for visual comparison. The *y*-axis represents the relative peak intensity of the metabolite fragments at their respective retention times. The chromatograms show some time-dependent variations (indicated with the red circles), providing an indication to the variations in the individual metabolomes.

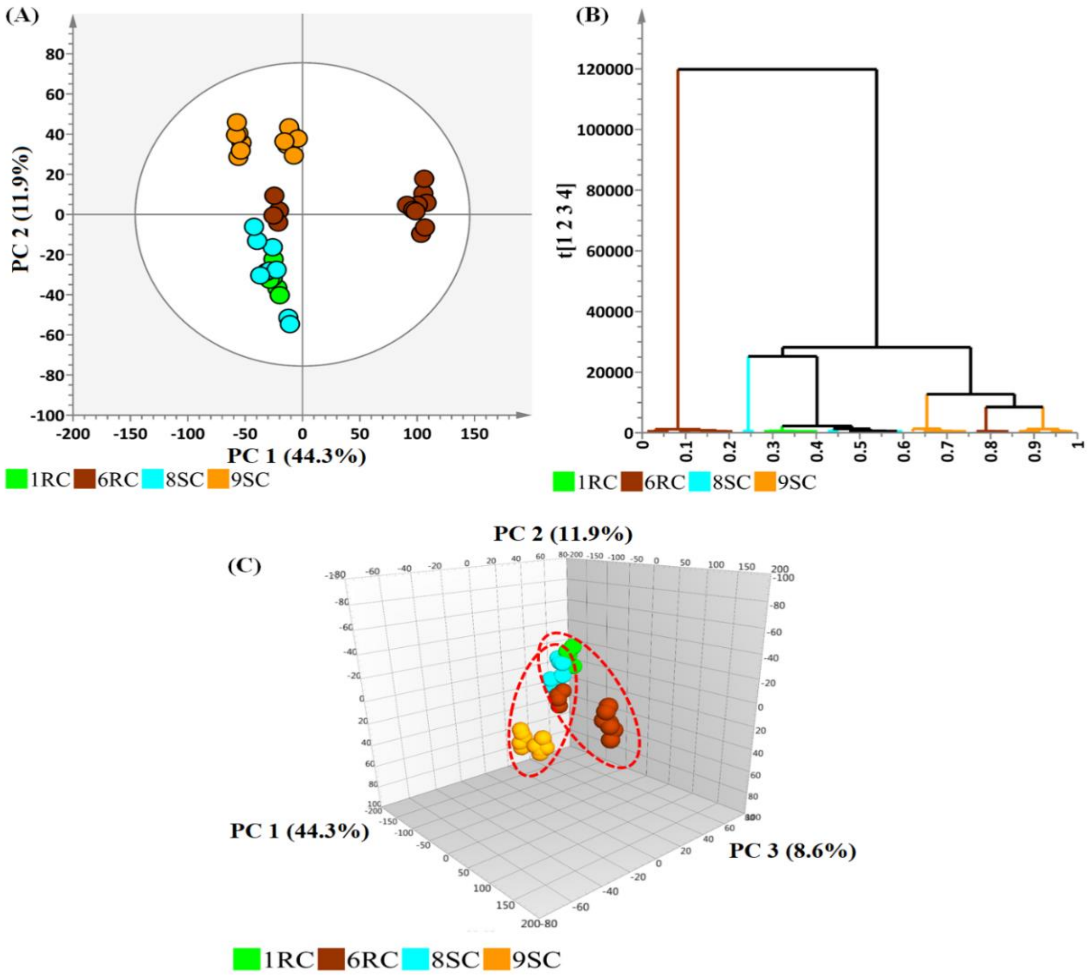

Figure S3. PCA score plot indicating the general grouping of the variables in the data sets for <u>stem</u> extracts. 1RC, 6RC, 8SC and 9SC specify the four cultivars differing in resistance to *Ralstonia solanacearum*, with 1RC being highly resistant and 8SC exhibiting intermediate resistance. **(A)** The 2D PCA plot of the LC-MS data, from the four cvs, illustrates the general clustering of the variables. The scores plot was computed using the first two principal components (PC1 *vs*. PC2). The circle in the score plot represents Hoteling's T2 with 95% confidence interval. **(B)** The HCA plot complimented the information provided by the PCA plots by providing the inter-relationships between the four cvs. The HCA plot also gives an indication that the samples could be separated into two major groups (9SC *vs*. 1RC, 6RC, 8SC) along with a further separation of the second hierarchical clustering (8SC *vs*. 1RC, 6RC). **(C)** The 3D PCA plot, with the data analysed in the first three principal components (PC1 *vs*. PC2 *vs*. PC3), allows an alternative and clearer view of the group clustering between the cvs. The red dotted lines indicates the clustering of the cvs.

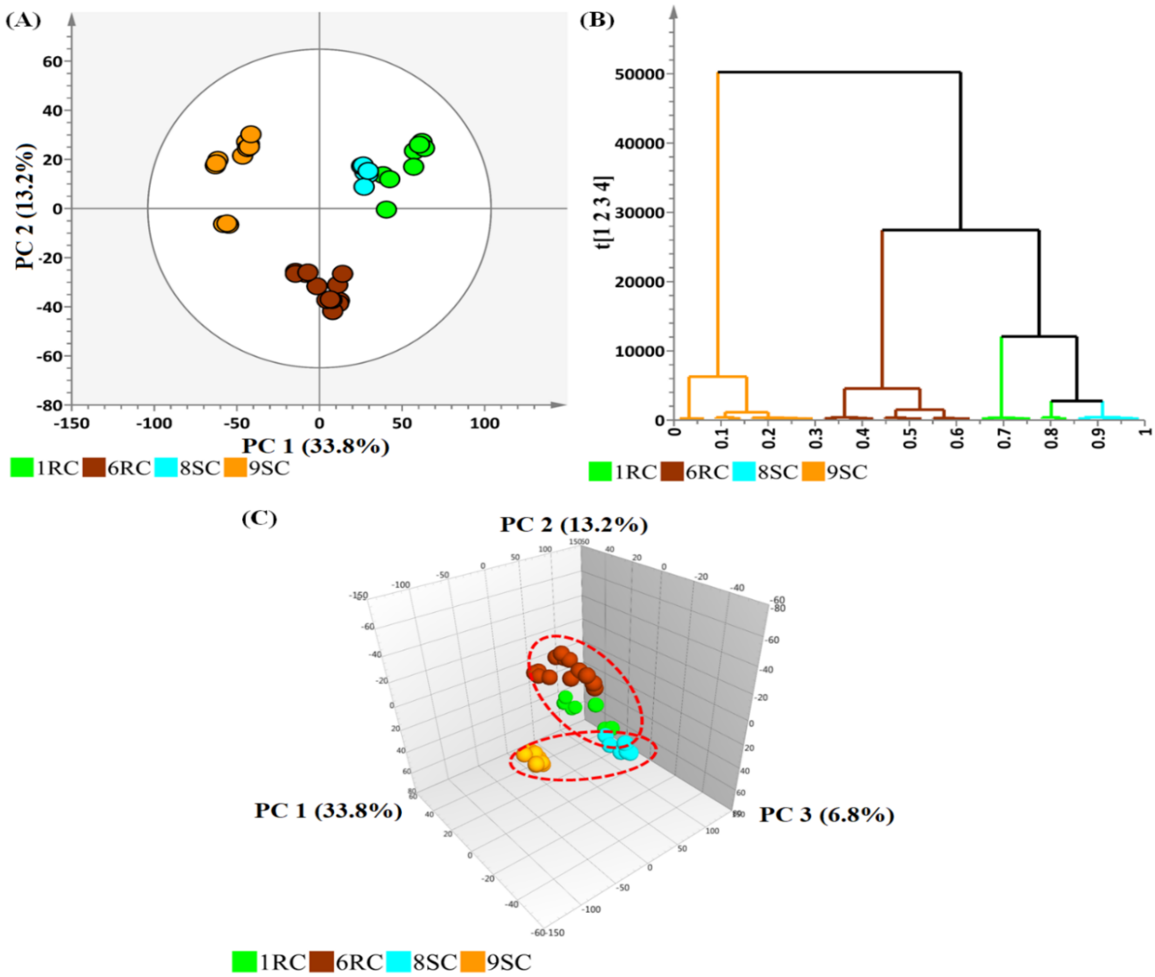

Figure S4. PCA score plot indicating the general grouping of the variables in the data sets for <u>root</u> extracts. 1RC, 6RC, 8SC and 9SC specify the four cultivars differing in levels of resistance towards *Ralstonia solanacearum*. **(A)** The 2D PCA plot of the LC-MS data, from the four cultivars, illustrates the general clustering of the variables. The scores plot was computed using the first two principal components (PC1 *vs*. PC2). The circle in the score plot represents Hoteling's T2 with 95% confidence interval. **(B)** The HCA plot complimented the information provided by the PCA plots by providing the inter-relationships between the four cvs. The HCA plot also gives an indication that the samples could be separated into two major groups (9SC *vs*. 1RC, 6RC, 8SC) along with a further separation of the second hierarchical clustering (8SC *vs*. 1RC, 6RC). **(C)** The 3D PCA plot, with the data analysed in the first three principal components (PC1 *vs*. PC2 *vs*. PC3), allows an alternative and clearer view of the group clustering between the cvs.

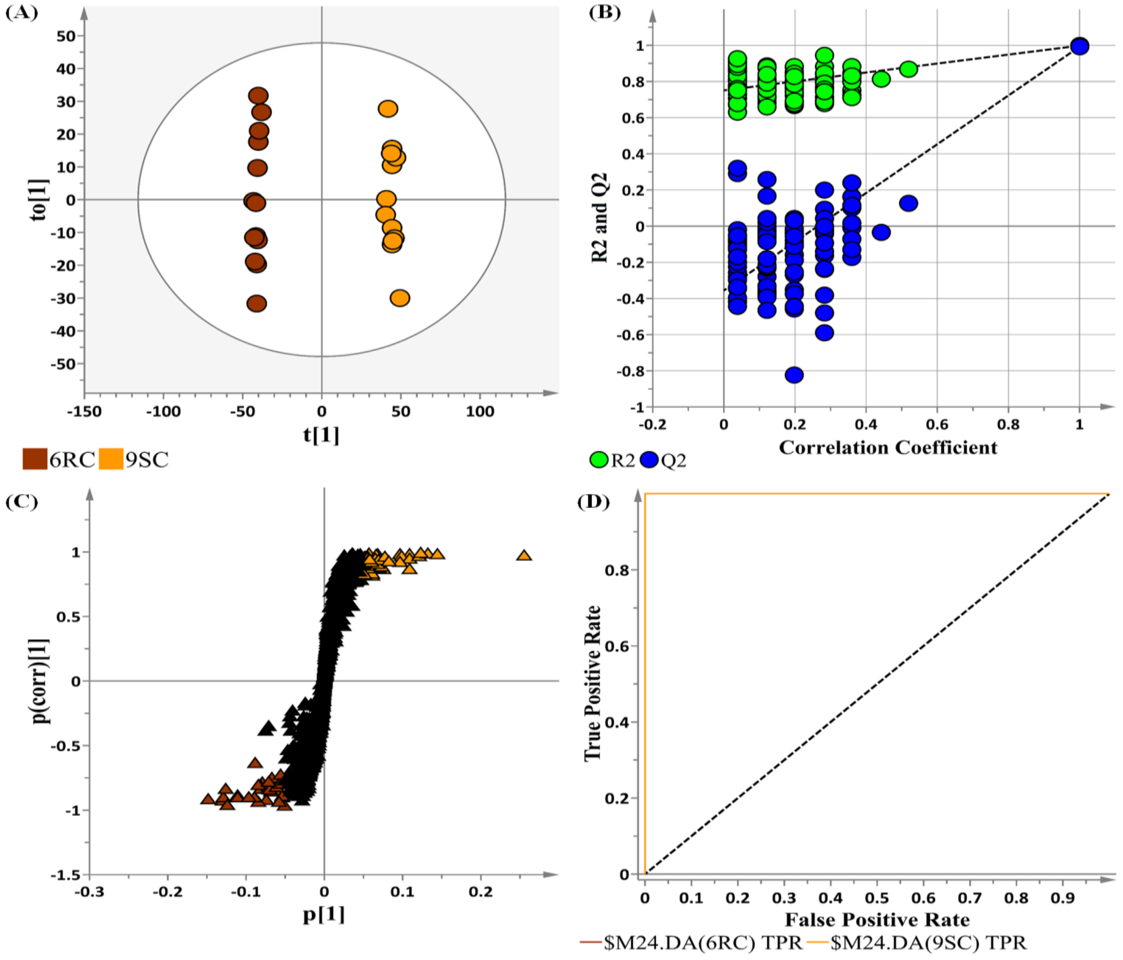

Figure S5. OPLS-DA model for the data processing for comparative analysis of extracts from leaf tissues of the 6RC (brown) and 9SC (orange) cultivars. (A) An OPLS-DA score plot summarizing the relationship among different datasets to visualize group clustering between the two cvs. (B) The response permutation test plot (n = 100) for the OPLS-DA model. (C) An OPLS-DA loading S-plot. (D) A receiver operating characteristic (ROC) plot summarizing the ability of the binary classifier.

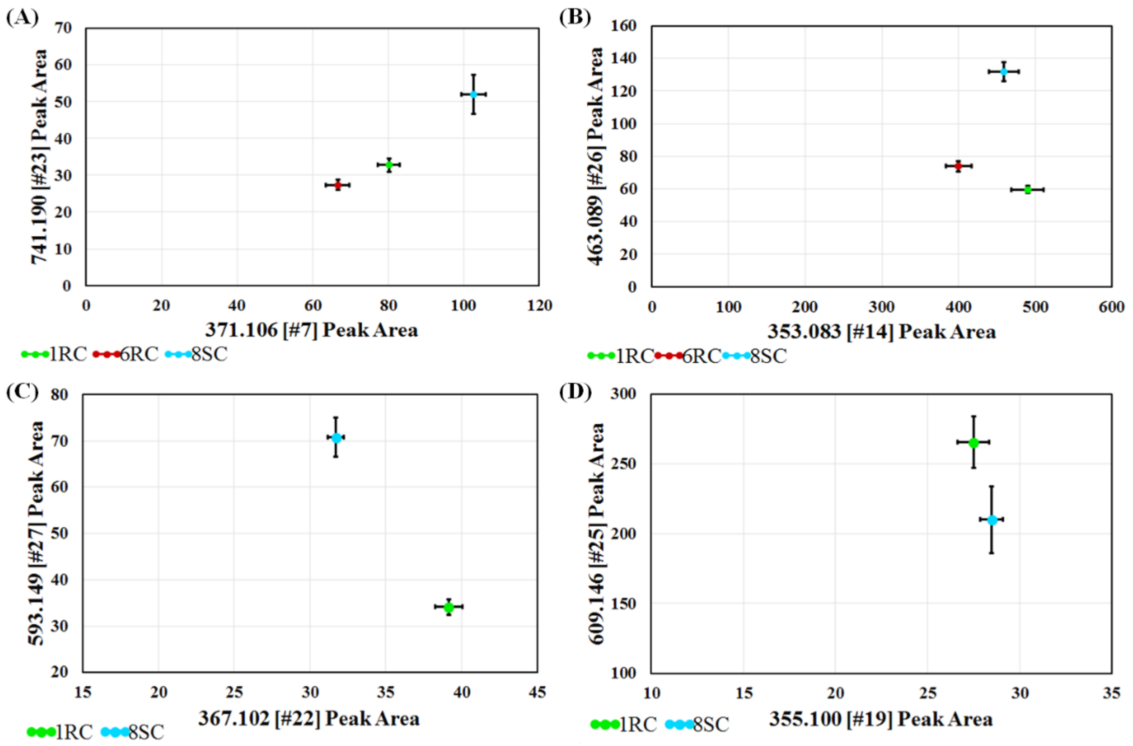

Figure S6. The relative quantification of annotated metabolites representative of different metabolic pathways in extracts from leaf samples of the following cultivars: 1RC (green), 6RC (red) and 8SC (light blue). (A) The peak area of [#7] *m*/*z* 371.059 plotted against that of [#23] *m*/*z* 741.190 for 1RC (green), 6RC (red) and 8SC (light blue). (B) The peak area of [#14] *m*/*z* 353.083 plotted against that of [#26] *m*/*z* 463.089 for 1RC (green), 6RC (red) and 8SC (light blue). (C) The peak area of [#22] *m*/*z* 367.102 plotted against that of [#27] *m*/*z* 593.149 for 1RC (green) and 8SC (light blue). (D) The peak area of [#19] *m*/*z* 355.100 plotted against that of [#25] *m*/*z* 609.146 for 1RC (green) and 8SC (light blue). The relative quantification estimation of the metabolites was based on the integrated peak areas of the selected ions, along with the addition of standard deviation error bars.
